# The relationship between microclimate factors and fruit quality in different tree canopies of Xiahui No. 8 peach trees

**DOI:** 10.3389/fpls.2025.1551110

**Published:** 2025-06-05

**Authors:** Junquan Zhen, Yuke Wang, Hening Xia, Hui Li, Haixia Wu, Caiping Zhao, Dong Wang

**Affiliations:** ^1^ College of Mechanical and Electronic Engineering, Northwest A&F University, Yangling, China; ^2^ Key Laboratory of Agricultural Internet of Things, Ministry of Agricultural and Rural Affairs, Yangling, China; ^3^ Shaanxi Key Laboratory of Agricultural Information Perception and Intelligent Service, Yangling, China; ^4^ College of Horticulture, Northwest A&F University, Yangling, China

**Keywords:** peach, canopy, microclimate, fruit quality, Xiahui No. 8

## Abstract

In this study, 6-year-old Xiahui No. 8 peach trees were used to investigate the effects of microclimates on their fruit quality between open-center and Y-shaped tree canopies. The impacts of the two canopy structures on light distribution, temperature, and relative humidity (RH) within the canopy, and their correlation with fruit quality metrics such as weight, hardness, soluble solid content (SSC), and dry matter content (DMC) were analyzed. The open-center-shaped trees had more favorable light distribution, with higher light intensity in the upper canopy layers and a larger light interception area, leading to improved fruit yield and quality compared to Y-shaped trees. The open-center-shaped trees also had higher single fruit weight, percentage of large fruits, SSC, flesh DMC and fruit hardness. However, free acidity showed only a minor difference between the two tree shapes. The study also found significant differences in the fruit coloration across canopy layers, with fruits in the upper layers exhibiting better coloration and higher SSC. Light intensity ranged from 30–90% and was strongly and positively correlated with fruit quality, thereby influencing the fruit size, coloration, and sugar-acid ratio. Overall, the open-center-shaped trees prove to be more conducive to improving fruit quality and yield. Nevertheless, proper management of the tree shapes, branch distribution, and light intensity is essential in optimizing fruit quality and maximizing the economic benefits of peach orchards.

## 1 Introduction

The canopy microclimate is influenced by the surrounding atmospheric climate and the growth and development of the tree. Hence, a larger crown volume and greater leaf area significantly affect the microclimate (Yang et al., 1998). Since the 1960s, most research on the leaf canopy microclimates has been concentrated abroad (Jackson, 1970; Palmer and Jackson, 1977; Barritt, 1988; Iglesias and Echeverria, 2022). These studies focused on the impacts of canopy structure on the physiological ecology of fruit trees and their economic benefits and differences in microclimates under different canopy shapes and their effects on fruit quality and yield. In China, experimental studies on fruit tree leaf canopy microclimates began in the 1970s. The concept of leaf canopy microclimate was first introduced by Zhang (1989) in their research on the influence of leaf canopy microclimates on grapefruit quality and yield. Since then, extensive studies have been conducted domestically and internationally on the relationships among leaf canopies, light energy, microenvironment assessment, microclimate differences, and the effects of canopies on fruit quality and yield (Dussi et al., 2005; Feng et al., 2014; Niu et al., 2015; Jia et al., 2016).

In recent years, research on leaf canopy microclimates in fruit trees such as peaches, apples, and pears has made significant progress. For instance, light is more uniformly distributed in the central leader shape of peach trees than in their Y-shaped, while its intensity in open-center (or open vase), Y-shaped and main trunk canopies increases from the bottom to the top and from the inside to the outside (He, 2007). In apple trees, the light intensity in the open-center canopy is the highest (Su, 2008; Wang et al., 2011). Additionally, the differences in temperature and humidity in the outer canopy of the Korla fragrant pear are greater than those in the middle (Niu et al., 2015).

Open-center systems capture more light, while Super High-Density systems have higher resource efficiency (Casanova-Gascón et al., 2019). Significant advances have also established the 3D models of fruit tree canopies, light interception, factors affecting photosynthesis, and the photosynthetic characteristics of canopies (Tang et al., 2015; Rojo et al., 2023).

The formation of fruit quality involves a series of physiological and biochemical processes, including carbohydrate metabolism, changes in sugar and acid content (AC), and the formation of dry matter. Studies have shown that the SSC, AC, DMC are key indicators for evaluating fruit quality (He et al., 2023). Biochemical analyses of different peach cultivars by Bassi and Selli (1990). indicated that the composition of sugars and acids is a core factor determining flavor quality. Furthermore, Tsipouridis and Thomidis (2005) confirmed that rootstock type significantly affects the absorption of mineral nutrients in fruits, thereby regulating SSC and AC levels. In addition to intrinsic physiological factors, various pre-harvest factors such as differences in microclimate, tree vigor, and leaf area can impact fruit quality (Minas et al., 2018). The differences in canopy position and microclimate also affect the fruit quality and significantly influence the development process and yield. Factors such as leaf light energy utilization efficiency, source-sink distance, branch positioning, and hormone signaling contribute to the canopy position of peach fruits, and impact their intrinsic quality (Anthony et al., 2021).

Improving fruit quality and yield is a key focus in fruit tree research. While previous studies have explored the relationship between tree shape, light, and fruit quality, there is relatively less research on the relationships among microclimates of different peach tree shapes, branch compositions, and fruit quality, yield, grading, and economic benefits (Li et al., 2013; Feng et al., 2014; Rudell et al., 2017; Serra et al., 2018; Minas et al., 2021). These studies have also revealed that tree shape and canopy structure significantly affect light distribution within the canopy, which in turn influences fruit growth and quality. Additionally, certain branch compositions are found to optimize fruit exposure to light, leading to improved yield and fruit grading. However, there is a lack of comprehensive studies examining the combined effects of canopy microclimates and branch distributions on the overall economic performance of fruit orchards. Therefore, an in-depth study of the microclimates and branch compositions of different tree shapes and canopies can aid in establishing efficient tree shapes, reveal the relationships between canopy microclimates and branch distributions, improve fruit quality and yield, and help in the analysis of cost-benefit ratios.

## 2 Materials and methods

### 2.1 Experimental materials

The experiment was conducted at the horticultural experimental garden of Northwest Agriculture and Forestry University in Yangling Demonstration Zone, Shaanxi Province (34°17’50.7”N 108°04’07.6”E). The experimental site consisted of sandy loam with 67.45 mg/kg of nitrogen (N), 140.73 mg/kg of phosphorus (P), 307.00 mg/kg of potassium (K), 20.12 g/kg of organic matter, and pH 7.93 in the 0−20 cm soil layer. The open-center training system facilitates light penetration and ventilation within the central canopy, which helps enhance fruit quality (Szewczuk and Gudarowska, 2012). The Y-shaped training system makes efficient use of vertical space and improves light exposure in the upper canopy (Sobierajski and Blain, 2022). These advantages make the two training systems ideal for studying how tree structure affects canopy microclimate and fruit quality. Therefore, in this study, six-year-old Xiahui No. 8 peach trees were trained using two different systems: the open-center system with a spacing of 3 m × 4 m, and the Y-shaped system with a spacing of 2 m × 4 m. For each training system, 4 trees were selected as experimental subjects. Routine horticultural care (pruning, thinning, irrigation, fertilization, and pest control) was applied to all trees throughout the season. In addition, fruit from both training systems was thinned approximately 30 days after full bloom by leaving about one fruit every 20 cm of shoot.

### 2.2 Measurement of canopy microclimate

**Figure 1:**
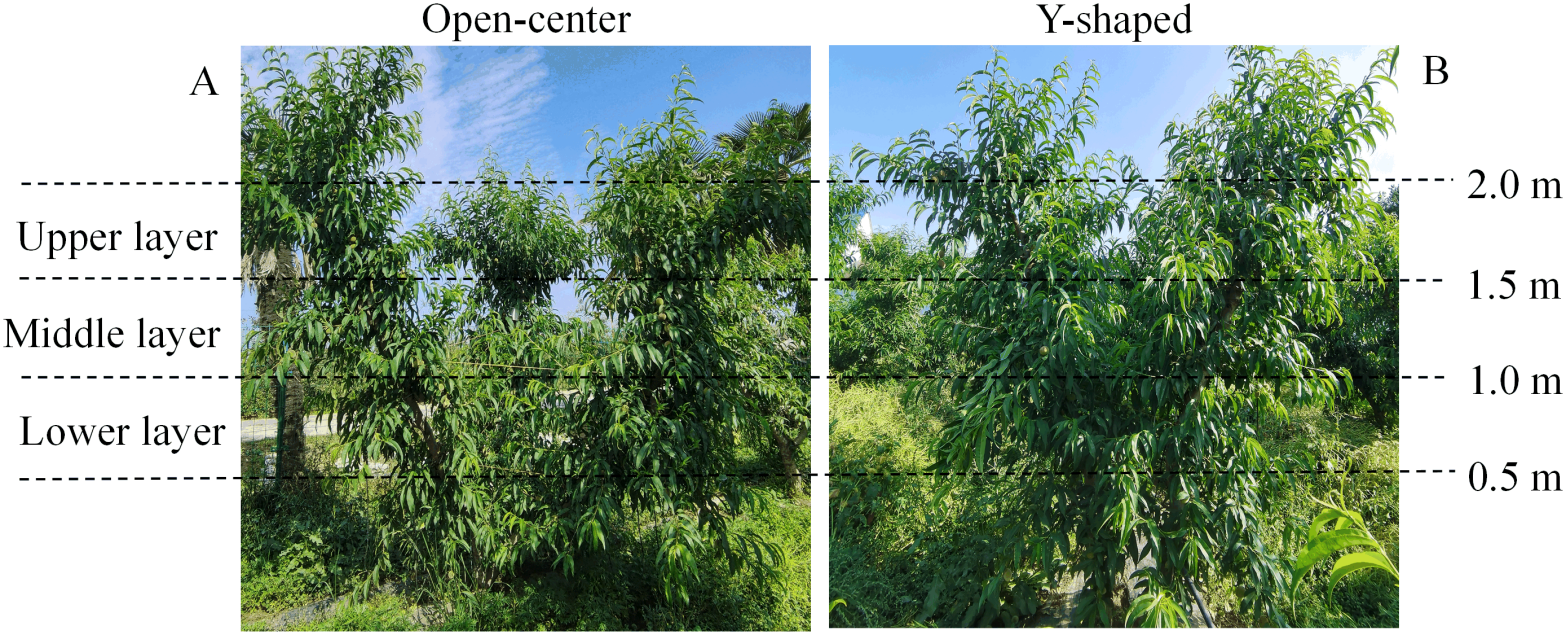
Peach trees with open-center **(A)** and Y-shaped **(B)** canopy layers.

The microclimate probes were vertically installed at the Lower (0.8 m), Middle (1.3 m), and Upper (1.8 m) parts of the open-center and Y-shaped peach canopies from the base of the trunk (**Figure 1**) and uniformly oriented towards the northeast, with two probes for each main branch at each canopy level. Afterwards, temperature, humidity, and light intensity were automatically recorded every 10 minutes (Liu et al., 2022).

### 2.3 Measurement of leaf area, chlorophyll, and dry matter content

Fifteen leaves from each of the three layers of each main branch were collected and their leaf area was measured using a leaf area meter (LI-3100C, LI-COR, Lincoln, NB, USA). The leaves were then punched into small discs using a hole punch and 0.1 g was mixed with 10 ml of 80% acetone (v/v) prepared with acetone and distilled water in a ratio of 4:1 in 10 ml centrifuge tubes. The preparation for each treatment was carried out in triplicates. The reaction mixtures were kept in the dark for 24 hours and occasionally shaken to ensure complete extraction and then used to measure chlorophyll a (*Ca*) and chlorophyll b (*Cb*) contents. A microplate was prepared, and 150μl of the extract was added to each well. The absorbance at 663 and 645 nm was measured using a microplate reader (Model-3550, Bio-Rad, Hercules, CA, USA). The formulas: *Ca* = 12.7A_663_ - 2.69A_645_ and *Cb*= 22.9A_645_ - 4.68A_663_ were used to calculate *Ca* and *Cb* contents (Porra, 2002; Chen et al., 2003), where A_663_ and A_645_ represent absorbance at 663 and 645 nm, respectively.

To measure the dry matter content (DMC), the above-remaining leaves were quickly chopped, mixed and then ground in a High-speed Tissue Homogenizer (TISSUELYSER-24L, Jingxin, Shanghai, China) for 1−2 minutes. A 2−5 g sample was weighed and dried in a dried centrifuge tube at 70°C for 4 hours. The weight of the samples was then recorded to an accuracy of 0.0002 g. The sample was further dried for 1 hour, and the weight was recorded again until the difference between the two weights was no more than 0.001 g to indicate a constant weight. The DMC of the leaves was calculated using the formula: DMC (%) = [(M2 - M0)/(M1 - M0)] * 100%, where M0 is the weight of the empty dried centrifuge tube, M1 is the weight of the dried centrifuge tube plus the sample, and M2 is the weight of the dried centrifuge tube plus the sample at constant weight (Shipley and Vu, 2002).

### 2.4 Measurement of daily photosynthetic variation and photosynthetically active radiation

To assess the daily photosynthetic variation, the net photosynthetic rate (Pn), transpiration rate (E), intercellular carbon dioxide concentration (Ci), and stomatal conductance (Gs) of leaves were measured at 8:00, 10:00, 12:00, 14:00, 16:00, 18:00, and 20:00 hours at 1.8, 1.3 and 0.8 m of the trunk. To evaluate the light distribution at different canopy levels and understand how light availability influences the photosynthetic capacity of leaves, the PAR at the upper, middle and lower canopy layers was determined by measuring light intensity using an integrated light meter (TA8121, Tasi, Suzhou, China) at heights of 1.8, 1.3 and 0.8 m. The PAR was then calculated using the formula, PAR = light intensity * 0.0185, where 1 Lux = 0.0185 μmol·m^-^²·s^-^¹ for sunlight (Thimijan and Heins, 1983; Ge et al., 2023).

### 2.5 The branch composition analysis

Branches on peach trees were measured using a 1.0 m ruler. The branches were then categorized as bouquet fruiting branches with height ≤5 cm, short fruiting branches with heights ranging from 5 to < 15 cm, medium fruiting branches with heights between 15 and 30 cm, long fruiting branches between 30 and 60 cm, and excessive growth fruiting branches measuring more than 60 cm. The number of each branch type was counted at different canopy levels, including the upper layer located between 1.5 and 2 m, the middle layer at 1−1.5 m, and the lower layer found from 0.5−1 m.

### 2.6 Fruit quality analysis

Once the fruits reached commercial maturity (with firmness ranging from 40N to 60N), they were harvested simultaneously from different canopy levels. All fruits were carefully labeled for subsequent analysis. 15 fruits were randomly selected from each canopy level for quality assessment. Each fruit was weighed individually using a high-precision balance to determine its single fruit weight. A fruit hardness tester (GY-4 with a 7.9 mm probe, Top, Zhejiang, China) was used to measure fruit hardness, which provided reliable readings for fruit firmness. The skin color of the fruits was assessed using a colorimeter (CR-400, Konica Minolta, Tokyo, Japan), which provided L, a, and b color values, offering insights into the ripeness and visual appeal of the fruit. The soluble solids content (SSC), an important indicator of fruit sweetness, was measured using a refractometer (PR-101α, Atago, Tokyo, Japan), following standard protocols for refractive index measurement, while the AC, was quantified using an acidity tester (PAL-BXIACID5, Atago, Tokyo, Japan). This device accurately measures the total AC in the fruit’s juice, indicating its tartness. The DMC of the fruit flesh was determined as described in Section 2.3, by ensuring consistency and accuracy across all measurements. This measurement gives an indication of the fruit’s solid content and texture, which is important in determining its quality. We also, estimated the fruit yield at 10 CNY/kg, and used the value for a cost-benefit analysis to assess the economic output of different tree shapes in the study.

### 2.7 Fruit grading standards

The fruit grading standards were based on the Jiangsu Province Xiahui No. 8 peach grading standard DB32/T 2594-2013 (Zhang et al., 2023a), where fruits weighing ≥250.0 were indicated as premium, ≥220.0 g as primary, ≥180.0 g as secondary and <180.0 g as substandard.

### 2.8 Data analysis

Raw data were organized, processed, and visualized using Microsoft Excel 2021 and Origin 2019. The spatial distribution maps were created using MATLAB 2019a. One-way analysis of variance and linear regression analysis were conducted with SPSS 2021, at a significance level of α = 0.05.

## 3 Results

### 3.1 Differences in microclimate and spatial distribution in different open-center and Y-shaped canopies

#### 3.1.1 The differences in the microclimate

**Figure 2:**
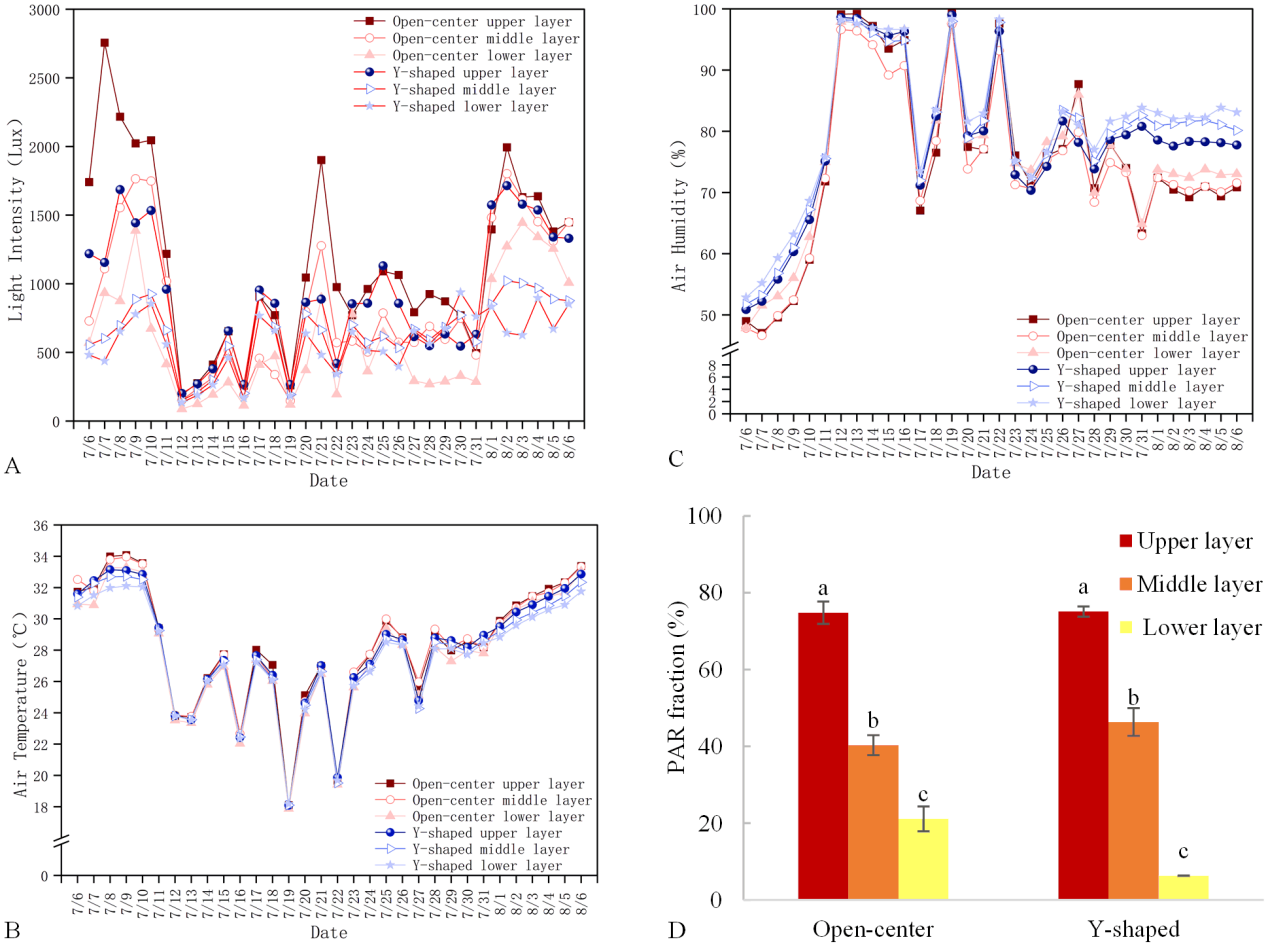
Microclimate changes from July 6 to August 6 in open-center and Y-shaped canopies: **(A)** Light intensity; **(B)** Air temperature; **(C)** Air humidity; **(D)** PAR proportion.

Generally, the light intensity (**Figure 2A**) and air temperature (**Figure 2B**) in the Open-center canopy were slightly higher than those in the Y-shaped canopy across the Upper, Middle, and Lower layers from July 6 to August 6. Both training systems exhibited a consistent vertical distribution pattern, with light intensity and temperature decreasing from the Upper to the Lower layers (Upper > Middle > Lower). In contrast, air humidity showed the opposite trend (**Figure 2C**), with the Y-shaped canopy maintaining higher humidity across all layers. Additionally, humidity increased progressively from the Upper to the Lower layers in both canopy structures (Upper < Middle < Lower). Both training systems received the highest PAR in the Upper layers and the lowest in the Lower layers, with the Lower-layer PAR of the Y-shaped canopy significantly lower than that of the Open-center canopy (**Figure 2D**).

#### 3.1.2 Spatial distribution of microclimate

**Figure 3:**
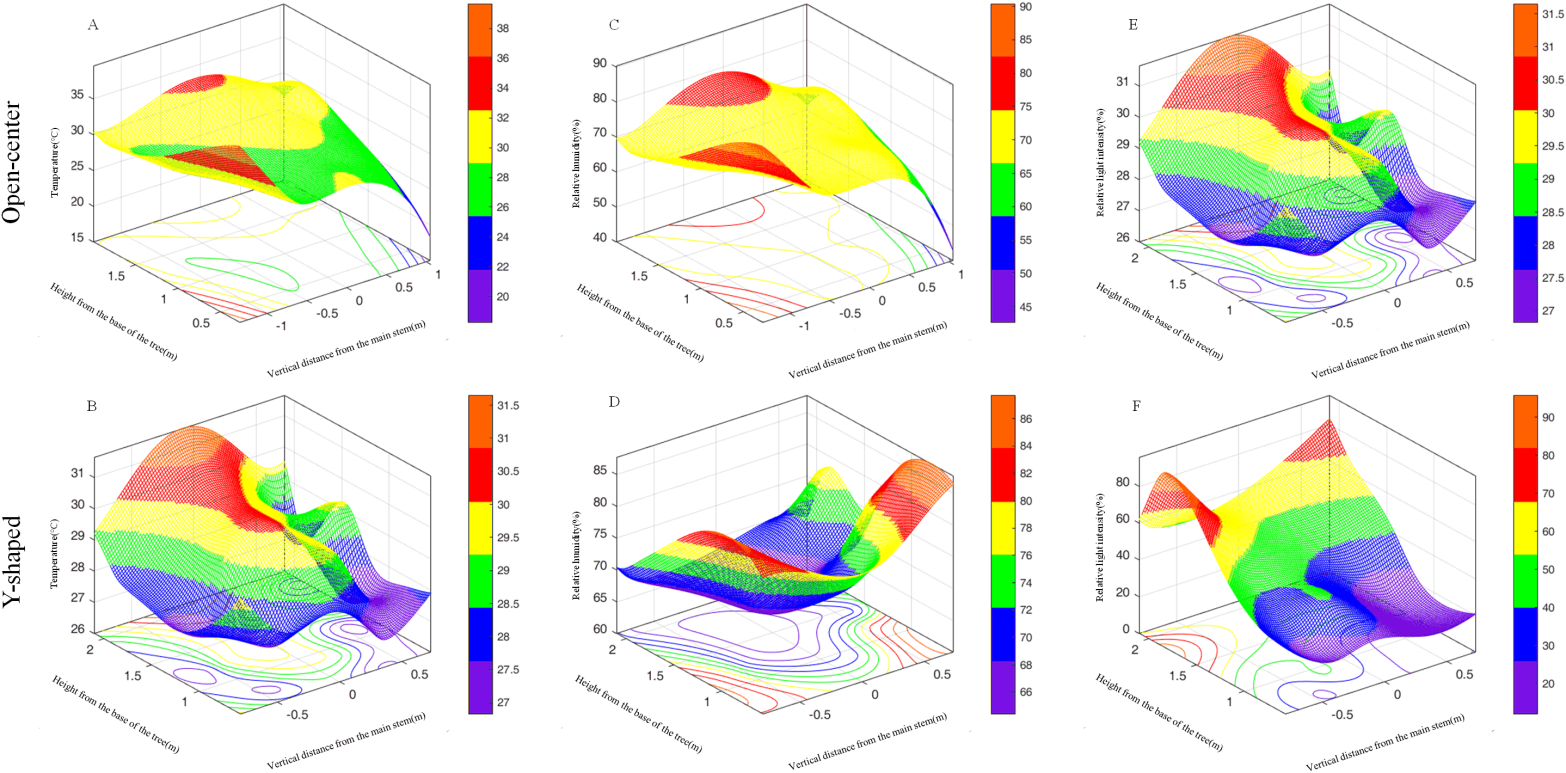
Spatial distribution changes of microclimate factors in open-center and Y-shaped canopies: (**A, B**: Temperature; **C, D**: Relative humidity; **E, F**: Relative light intensity).

A lateral comparison of temperatures in the open-center (**Figure 3A**) and Y-shaped (**Figure 3B**) found lower temperatures closer to the trunk. Vertically, the temperature in the canopy progressively decreased from top to bottom, with little variation in the same canopy layer. In the open-center trees, the average temperature ranged from 29.20 to 29.76°C in the upper layer, 28.92 to 29.31°C in the middle layer, and from 28.64 to 28.94°C in the lower layer. In the Y-shaped trees, the average temperature ranged from 27.39 to 29.52°C in the upper layer, 26.99 to 29.26°C in the middle layer and from 26.91 to 29.19°C in the lower layer. Therefore, the temperature distribution in the open-center canopy was more uniform and stratified than in the Y-shaped canopy. Laterally, higher relative humidity (RH) was found closer to the trunk (**Figures 3C, D**), while vertically, the RH in the canopy progressively increased from the top to bottom, with little variation within the same layer. The average RH in the upper layer of the open-center trees ranged from 67.03 to 67.91%, 69.53 to 69.67% in the middle layer, and from 70.15 to 71.53% in the lower layer. In the upper layer of the Y-shaped canopy, the average RH ranged from 68.49−73.33%, 70.43−78.84% in the middle layer, and from 72.31−80.19% in the lower layer. The RH in the open-center canopy was also uniformly distributed and more stratified than those in the Y-shaped canopy. A lateral comparison of relative light intensity between open-center and Y-shaped canopies revealed lower relative light intensity closer to the trunk. Vertically, the relative light intensity in the canopy decreased from top to bottom, with little variation within the same canopy layer. In the open-center canopy, the average relative light intensity in the upper layer ranged from 66.80 to 95.06%, in the middle layer from 40.63 to 62.43%, and in the lower layer from 25.90 to 38.46%. In the Y-shaped canopy, the average relative light intensity in the upper layer ranged from 56.30 to 93.64%, in the middle layer from 17.49 to 41.20%, and in the lower layer from 20.61 to 38.92% (**Figures 3E, F**).

#### 3.1.3 The differences in branch composition and spatial distribution

The quantity of each branch type in the open-center tree was generally and significantly higher than those in the Y-shaped, with the open-center peach tree having 156 branches, while the Y-shaped had only 75 branches (**Table 1**), with more fruit branches generally found in the lower canopy. The study also showed that the distribution of fruit branches in the canopy of the open-center peach tree is relatively uniform, with the highest number of short and medium fruit branches found in the lower and middle layers, respectively, while fewer branches copied the upper layers. The Y-shaped had more fruit branches in the upper layer and fewer in the lower layer, with the most medium branches found in the middle layer and the longest branches in both the upper and lower layers.

**Table 1:** Distribution of branches in different open-center and Y-shaped canopies.

Shape	Canopy	Bouquet (≤5 cm)	Short (5−15 cm)	Middle (15−30 cm)	Long (30−60 cm)	Leggy (>60 cm)	Total
Open-center	Upper	1 ± 1.0 b	10 ± 4.0 a	10 ± 1.0 c	22 ± 1.0 a	4 ± 1.0 a	47 ± 2.6 a
	Middle	8 ± 2.6 a	2 ± 1.0 b	13 ± 2.0 b	26 ± 1.0 a	4 ± 1.0 a	53 ± 12.3 a
	Lower	7 ± 1.0 a	10 ± 2.6 a	17 ± 7.8 a	17 ± 2.6 a	5 ± 2.6 a	56 ± 11.5 a
Y-shaped	Upper	0 ± 0.3 a	3 ± 1.0 a	8 ± 0.6 b	14 ± 1.2 a	2 ± 1.2 a	28 ± 1.5 a
	Middle	1 ± 0.0 a	4 ± 1.0 a	12 ± 0.3 a	8 ± 0.3 b	1 ± 0.3 a	26 ± 2.0 ab
	Lower	2 ± 1.0 a	4 ± 0.3 a	5 ± 0.3 c	8 ± 1.0 b	2 ± 1.0 a	21 ± 1.7 b

Different letters indicate significant differences (p < 0.05).

### 3.2 Differences of leaf mass and photosynthetic characteristics in different open-center and Y-shaped canopies

#### 3.2.1 Differences in leaf area and chlorophyll content

**Figure 4:**
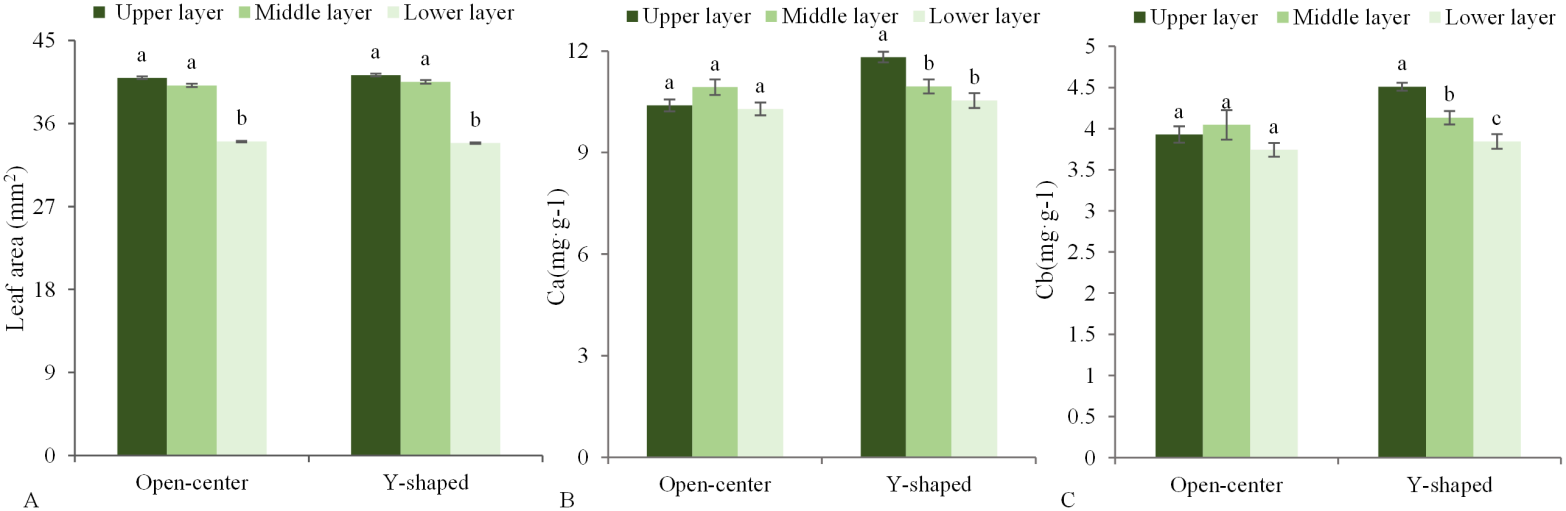
Difference in leaf area **(A)**, *Ca* **(B)**, and *Cb* **(C)** in different open-center and Y-shaped canopies. Different letters indicate significant differences (p < 0.05).

The leaf area between the open-center and Y-shaped tree canopies did not differ significantly but generally increased from the lower to middle and upper layers in each tree shape. The leaf area of the middle and upper canopy layers was significantly larger than that of the lower canopy layer (**Figure 4A**). The Y-shaped canopy showed a more pronounced variation in chlorophyll content. Therefore, the *Ca* and *Cb* contents in the upper canopy were significantly higher than those found in the middle layer. However, the *Ca* content insignificantly differed between the middle and lower layers, while the *Cb* content in the middle layer was significantly higher than those in the lower layer (**Figure 4B**). In the open center, there was no significant difference in chlorophyll content among the canopy layers (**Figure 4C**).

#### 3.2.2 Differences in photosynthetic characteristics

**Figure 5:**
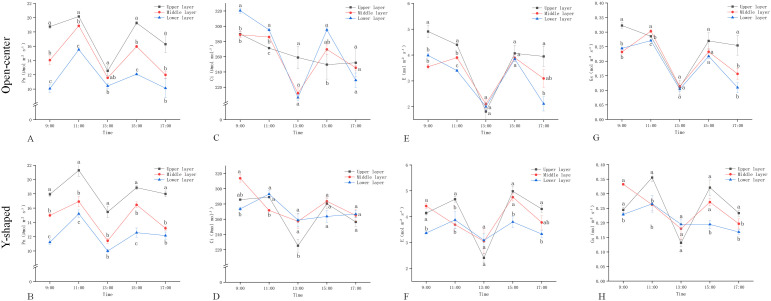
Diurnal variation of photosynthetic parameters of leaves in different open-center and Y-shaped canopies: (**A, B**: *Pn*; **C, D**: *Ci*; **E, F**: *E*; **G, H**: *Gs*).

The results indicated a consistent trend in the daily *Pn* changes for both tree shapes, with more *Pn* in the upper layer followed by those in the middle and lower layers (**Figure 5**). The *Pn* increased from 9:00 to 11:00 AM and then from 1:00 to 3:00 PM, with the highest day value at 11:00 AM. However, it decreased from 11:00 AM to 1:00 PM, and from 3:00 to 5:00 PM, with the lowest *Pn* value at 1:00 PM for both tree shapes, except for the lower layer of the open-center. The *Ci*, *E*, and *Gs* trends in both tree shapes were consistent with the *Pn* trends, except for the upper layer of the open center, where *Ci* continuously decreased from 9:00 AM to 5:00 PM, with the lowest values at 1:00 PM (**Figure 5**). However, as the temperature increased from 11:00 AM to 1:00 PM, the peach leaves entered a midday photosynthetic depression, causing their stomata to gradually close, thereby decreasing the *Gs*, *E*, *Ci* and *Pn*. Between 1:00 and 3:00 PM, the leaves recovered from the midday photosynthetic depression, and gradually regained their photosynthetic capacity. The most significant changes before and after the midday depression, from 11:00 AM to 3:00 PM, occurred in the upper layer of the Y-shaped, where *Ci*, *E*, and *Gs* dropped to levels below those of the lower layer. In contrast, the open center showed relatively stable performance (**Figure 5**).

### 3.3 Differences in peach fruit quality in different open-center and Y-shaped canopies

#### 3.3.1 Differences in fruit grading

**Figure 6:**
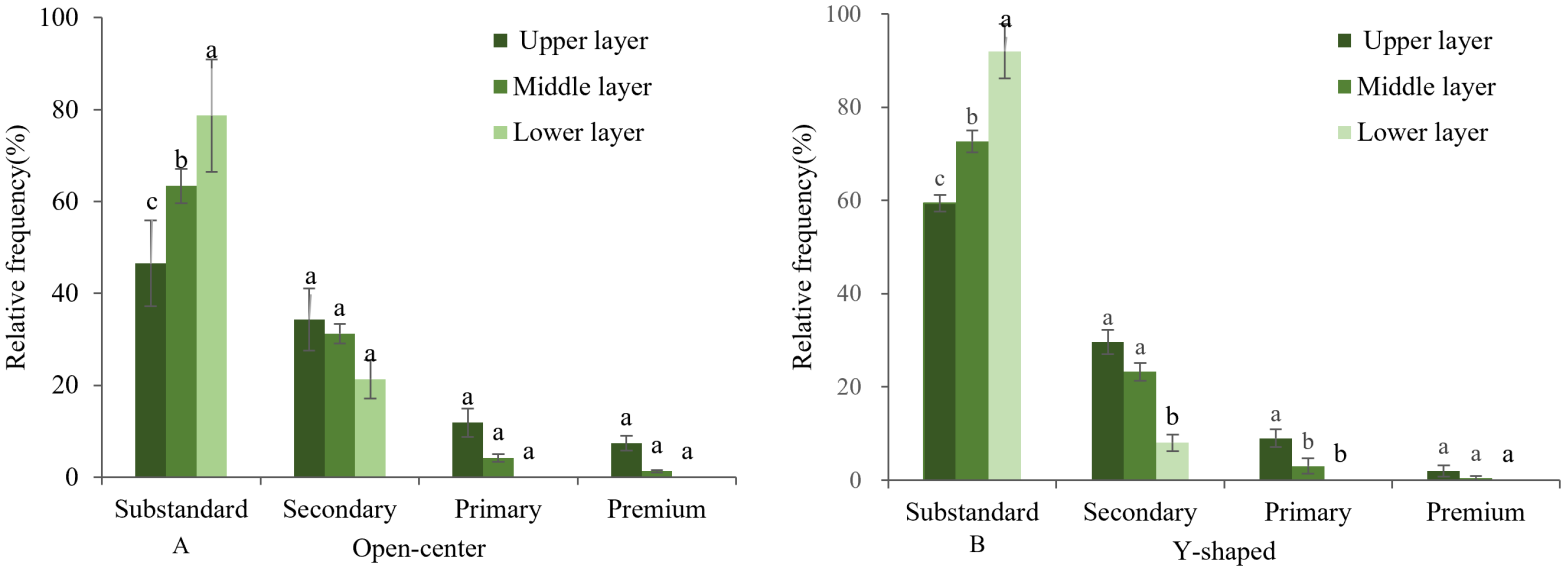
Fruit grading of peaches of open-center **(A)** and Y-shaped **(B)** canopies.

Most of the fruits found in the open-center and Y-shaped trees were substandard, followed by secondary, primary and premium fruits. The upper part of the canopy had the fewest substandard fruits, while the lower layer had the most. Premium and primary fruits were mainly distributed in the middle and upper parts of the canopy, with almost none in the lower part. On the other hand, the secondary, primary, and premium fruits were more uniformly distributed in the open center than in the Y-shaped. However, the proportion of substandard fruits in the Y-shaped was higher, with the fruits formed in the open center generally larger than those in the Y-shaped trees (**Figure 6**).

#### 3.3.2 Differences in fruit weight and hardness

As the peach fruit ripened, fruit weight and hardness differed between the open-center and Y-shaped trees across different leaf curtain layers (**Table 2**). The hardness of the fruits was lowest for both tree shapes at the upper parts of the canopy layers and higher in the lower and middle layers, with those in the Y-shaped harder than those in the open center. Fruits from both tree shapes showed the heaviest fruit weight at the upper parts of the canopy, with the lightest fruits located at the lower parts. Though there were insignificant differences in the fruit outputs and incomes between the different canopy layers of the two tree shapes, the higher yields in the open-center likely caused slightly higher profits than those in the Y-shaped peaches. Therefore, in peach production, it is important to promote the formation of high-quality fruits while ensuring yield to achieve better profits.

**Table 2:** Output and income of peaches in different open-center and Y-shaped canopies.

Shape	Canopy	Hardness/N	Single weight/g	Output kg/hm^2^	Income 10 K/hm^2^
Open-center	Upper	43.4 ± 2.1 b	186.7 ± 14.5 a	9640.7 ± 256.9 a	9.6 ± 0.3 a
	Middle	46.3 ± 2.4 b	167.9 ± 1.5 b	9493.9 ± 450.8 a	9.5 ± 1.5 a
	Lower	53.2 ± 2.2 a	144.9 ± 12.7 c	10119.5 ± 669.8 a	10.1 ± 0.7 a
	Total	47.6	166.5	29254.3	29.3
Y-shaped	Upper	48.1 ± 2.3 b	179.9 ± 2.2 a	9195.8 ± 650.1 a	9.2 ± 0.7 a
	Middle	52.0 ± 2.6 ab	160.2 ± 4.5 b	9678.4 ± 253.9 a	9.7 ± 0.3 a
	Lower	56.9 ± 1.6 a	134.6 ± 3.6 c	9822.7 ± 656.8 a	9.8 ± 0.7 a
	Total	52.3	158.2	28696.9	28.7

#### 3.3.3 Differences in skin color

Both the open-center and Y-shaped trees had brighter and redder skin on the upper layer with a significantly lower comprehensive color index value (h*) than those on the lower layer, making the colors of the upper layer fruits more vivid and bright. Generally, the skin color of the open-center fruits was brighter, redder, vibrant and saturated than those found in the Y-shaped (**Table 3**).

**Table 3:** Color change of peach peel in different Open-center and Y-shaped canopies.

Shape	Canopy	L*	a*	b*	C*	h*
Open-center	Upper	63.9 ± 1.0 a	28.4 ± 0.6 a	23.4 ± 0.2 b	36.9 ± 0.4 a	39.9 ± 0.8 c
	Middle	62.4 ± 0.7 a	24.0 ± 0.8 b	24.2 ± 0.2 a	34.6 ± 0.5 b	46.1 ± 1.2 b
	Lower	56.8 ± 0.7 b	21.0 ± 1.1 c	24.2 ± 0.3 a	32.7 ± 0.6 c	50.5 ± 1.8 a
Y-shaped	Upper	59.1 ± 0.7 a	26.5 ± 0.6 a	23.5 ± 0.2 b	35.7 ± 0.4 a	42.2 ± 0.8 b
	Middle	65.2 ± 0.6 ab	20.9 ± 0.6 b	23.7 ± 0.1 b	31.9 ± 0.4 b	49.5 ± 1.0 a
	Lower	78.5 ± 9.4 b	15.9 ± 1.0 c	24.7 ± 0.2 a	30.3 ± 0.4 c	53.8 ± 3.0 a

L* indicates the brightness of fruit surface color; +a* means red, –a* means green, +b* means yellow and -b* means blue; C* represents color saturation; H* represents the hue angle.

#### 3.3.4 Differences in soluble solids, acid and flesh DMC

**Figure 7:**
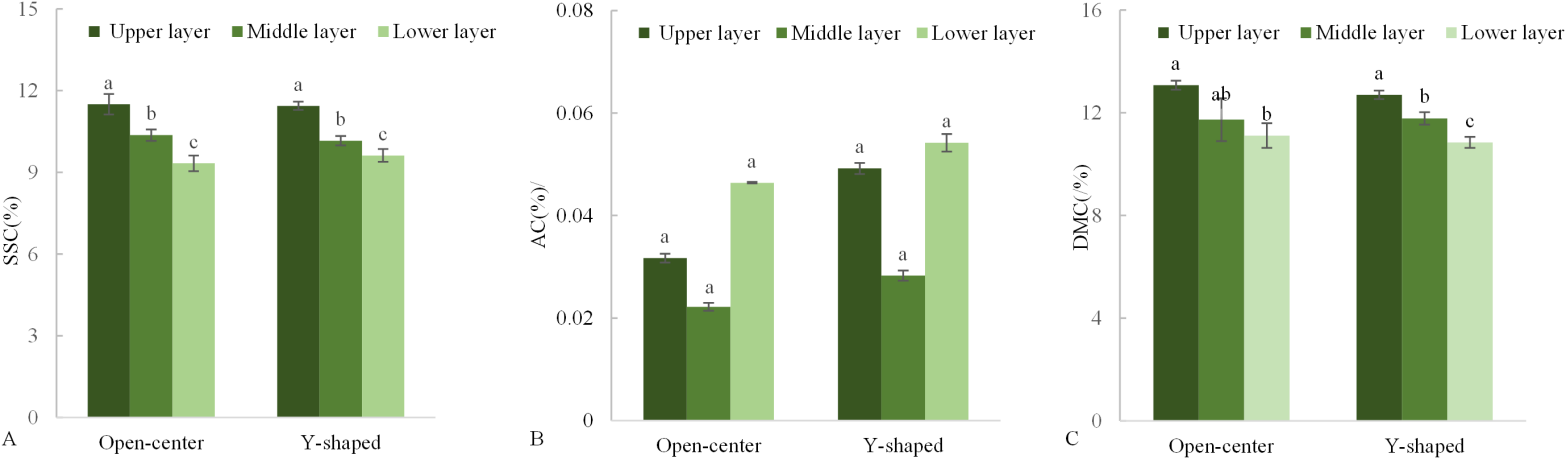
Changes in soluble solid content **(A)**, acid content **(B)** and dry matter content **(C)** in different open-center and Y-shaped canopies.

During the ripening period, Xiahui 8 peaches from both tree shapes exhibited the highest levels of SSC and flesh DMC in the upper canopy, with the lowest levels in the lower canopy. However, AC was insignificantly different across the different canopy layers (**Figure 7**) but was slightly higher in Y-shaped fruits than in open-center fruits, with both tree shapes showing the lowest acid levels in the middle parts of the tree.

### 3.4 Correlation between microclimates and fruit quality in open-center and Y-shaped canopies

The individual fruit weight and SSC were significantly and positively correlated with light intensity in both open-center and Y-shaped trees (**Table 4**). In the open-center fruits, the fruit hardness and AC significantly and negatively correlated with light intensity, while humidity was significantly and positively correlated with fruit hardness but significantly and negatively correlated with SSC. The DMC insignificantly correlated with temperature, humidity, and light intensity in the open-center fruits. In Y-shaped fruits, light intensity was significantly and positively correlated with DMC but significantly and negatively correlated with fruit hardness and AC. Humidity showed only a significant and negative correlation with DMC, while temperature had an insignificant correlation with all the fruit qualities in Y-shaped fruits.

**Table 4:** Correlation between microclimate and fruit quality.

Shape	Microclimate	Single fruit weight	Hardness	SSC	AC	DMC
Open-center	light	0.414**	-0.338*	0.527**	-0.324*	0.267
	humidity	-0.185	0.346*	-0.301*	-0.034	-0.152
	temperature	0.086	-0.221	0.085	0.131	0.213
Y-shaped	light	0.383**	-0.572**	0.494**	-0.389**	0.436**
	humidity	-0.071	0.131	-0.018	0.085	-0.294*
	temperature	0.032	-0.028	-0.03	-0.065	0.163

*0.05 level is significantly correlated, **0.01 level is significantly correlated.

Based on the correlation analysis results, further regression analysis was conducted on light intensity and fruit quality. The regression equations for single fruit weight, hardness, SSC, and AC with light intensity all reached significant levels, indicating that the established equations are reliable. The calculated values indicate that optimal relative light intensity values for improving the fruit quality of open-center canopies are higher than those for Y-shaped canopies (**Table 5**).

**Table 5:** Regression analysis of light intensity and fruit quality.

Shape	Quality	Regression equation	F	Optimum relative illumination value/%
Open-center	Single fruit weight	Y=1.5144X-0.0215X2 + 148.817	5.452**	35.22
	Hardness	Y=-0.0058X+0.0000421X2 + 44.972	5.355**	68.88
	SSC	Y=-0.0235X+0.00013X2 + 9.411	10.137**	90.38
	AC	Y=-0.0021X+0.00001201X2 + 0.076	3.324*	87.40
Y-shaped	Single fruit weight	Y=1.1025X-0.01576X2 + 125.975	8.464**	32.12
	Hardness	Y=-0.0998X+0.000869X2 + 56.514	17.314**	57.45
	SSC	Y=0.0298X-0.000173X2 + 8.163	13.989**	86.37
	AC	Y=-0.0013X+0.0000094X2 + 0.167	7.186**	69.15
	DMC	Y=0.021X-0.000127X2 + 10.834	8.986**	82.39

* means P < 0.05; ** means p < 0.01.

## 4 Discussion

### 4.1 Tree shape and microclimate distribution

Peach trees love light, and their shape and canopy structure directly affect the distribution of light within the canopy, branch and leaf growth, and the light interception rate, which affects fruit quality and yield (Génard et al., 2000; Farina et al., 2005; Anthony and Minas, 2025). In our study, we found that the light was vertically distributed more in the upper layer, followed by middle and lower layers but horizontally increased from inside to outside in both the open-center and Y-shaped canopy of Xiahui No. 8 peach trees. Meanwhile, light distribution in the Y-shaped canopy was more uniform compared to that in the open-center canopy, which is consistent with the findings of Yue et al. (2018) and Wen et al. (2019). The yield, including single fruit weight of the upper, middle, and lower layers of the open-center trees was slightly higher than that of the Y-shaped trees, while the fruits in all three layers of the open-center trees were less hard than the fruits in the Y-shaped trees. This indicates that the open-center canopy is more open, has a larger light-receiving area, and a higher light utilization rate. This is consistent with the results reported by Anthony et al. (2021) which showed that fruits growing under different light conditions experience variations in their maturation process and internal quality development, with fruit maturity increasing as light intensity increases.

The physiological processes of fruit trees, such as photosynthesis, respiration, and transpiration, need to occur under specific temperature and relative humidity (RH) conditions, which can affect fruit quality by regulating enzyme activity, as well as the SSC and DMC (Ou et al., 2023). In our study, the vertical distribution pattern of temperature in the canopy of both the open-center and Y-shaped trees was higher in the upper followed by the middle and lower layers, while horizontally the temperature gradually decreased from outside to inside. On the other hand, the distribution pattern of RH within the canopy was opposite that of the temperature.

### 4.2 Relationship between training system and fruit quality, yield and revenue

Different training system result in varying amounts and distributions of branches and leaves within the canopy, which alter its light intensity, temperature and humidity, directly impacting the fruit quality. Thus, the quality of fruits produced by different tree shapes varies (Zeng, 2022). The current study showed higher single fruit weight, percentage of large fruits, SSC, flesh DMC, and total number of fruiting branches in the open-center than in the Y-shaped trees. The primary reason for these differences may be that the open-center training system enables more efficient light interception and utilization, thereby enhancing photosynthetic activity, promoting the accumulation and transport of assimilates, and ultimately improving fruit DMC and SSC. These findings are consistent with those of Pieper et al. (2024) which emphasized that controlling the light environment within the canopy plays a crucial role in fruit quality development, with high light intensity being positively associated with superior fruit quality.

Moreover, significant differences in fruit surface coloration were observed among different canopy layers: fruits located in the upper canopy were notably redder and brighter than those in the middle and lower layers. This finding is consistent with the results reported Yue et al. (2018), primarily attributable to the higher photosynthetic intensity, extended light exposure duration, and elevated temperatures in the upper canopy, all of which promote the accumulation and synthesis of anthocyanins, resulting in redder and more vivid fruit coloration. Although the yield and revenue of different canopy layers between the two training systems showed no significant differences, the overall yield and revenue of open-center trees were slightly higher than those of Y-shaped trees. This is attributed to the fact that fruits from open-center trees exhibited higher sweetness, making them more appealing to consumers. This observation is consistent with the findings of Iglesias and Echeverría (2009) and Wen et al. (2019), which indicated that consumers generally prefer fruits with higher sugar content, thereby enhancing their market value.

Thus, the widely spaced main branches and numerous fruiting branch groups in the open-center peach tree, make it utilize light more efficiently than a Y-shaped tree, thereby enhancing its fruit quality.

### 4.3 Relationship between microclimate and fruit quality

The light intensity, RH and temperature inside the canopy significantly influence fruit quality and have complex interactions among themselves, with light intensity playing a dominant role (Zhang et al., 2019). In this study, we correlated light intensity, temperature and RH with single fruit weight, hardness, SSC, AC and flesh DMC, revealing a highly significant correlation between light intensity and the fruit quality attributes. Light intensity also significantly affects the coloration and sugar-acid ratio of fruit, thereby impacting its quality and yield. The study shows that fruits in the upper layers of the canopy have the greatest size, best coloration, and highest SSC. Additionally, the average temperature in the upper layers is higher than that in the middle and lower canopy, which promotes the activity of synthetic enzymes and accelerates the conversion and accumulation of sugars, ultimately leading to higher sugar content and elevated SSC levels in fruits from the upper canopy. These observations are consistent with previous studies on peach fruit composition and postharvest physiology, which similarly reported that enhanced light exposure and higher temperatures contribute to improved fruit coloration and increased sugar accumulation (Zhang et al., 2023b).

Overall, in the current study among the three major microclimatic factors, light intensity had the greatest correlation with fruit quality. Based on this, we established a binary linear regression equation between light intensity and fruit quality and calculated the optimal light intensity values for quality attributes. The optimal relative light intensity values between 30 and 90% enabled the Xiahui No. 8 peach to achieve high quality and yield as has been reported previously (Huang et al., 2015; Liu et al., 2021). Light intensity below 30% is characterized as low light efficacy, which is not conducive to producing high-quality and high-yield fruits.

## 5 Conclusion

This study explored the relationships between tree shape, microclimate, fruit quality, yield, and economic benefits in peach trees. The results showed that the open-centered canopy had higher light utilization, which in turn improved the single fruit weight, hardness, SSC, and DMC, and increased yield and revenue compared to the Y-shaped canopy. Furthermore, the number of fruiting branches within the canopy reflects the growth status and size of the tree, and maintaining a certain number of evenly distributed fruiting branches helps to effectively control fruit yield. The study also found that light intensity is the primary factor affecting fruit quality, with optimal relative light intensity ranging from 30 to 90%, which is also conducive to producing high-quality and yielding fruits. Therefore, proper management of tree shape, branch distribution, and light intensity plays a crucial role in improving fruit quality and economic benefits in peach orchards.

## Data Availability

The original contributions presented in the study are included in the article/supplementary material. Further inquiries can be directed to the corresponding author.
